# Inhalation of Food during Injection of Pentothal

**Published:** 1946

**Authors:** R. Hastings Moore

**Affiliations:** Honorary Assistant Anaesthetist, Bristol Royal Hospital; Honorary Anaesthetist and Lecturer in Analgesia, Bristol Maternity Hospital


### INHALATION OF FOOD DURING INJECTION OF PENTOTHAL

BY

R. Hastings Moore, M.B., Ch.B. M.R.C.S., L.R.C.P., D.A.

*Honorary Assistant Anaesthetist, Bristol Royal Hospital; Honorary Anaesthetist and Lecturer in Analgesia, Bristol Maternity Hospital.*

---

The accidents described below are of some importance, since pentothal is frequently administered in unfavourable conditions : if not promptly recognized they may prove fatal. During and after the invasion of France it was necessary to administer pentothal to hundreds of patients who had not been prepared and whose stomachs contained food. In four cases food was inhaled during the administration.

**Case 1.**—After a fourteen hours' session, at 3 a.m., while the last operation was in progress, the dental officer arrived to stitch up the mouth of a concussed and inebriated despatch-rider. There was free bleeding into the mouth : otherwise the man would have been evacuated. He objected to local anaesthesia and became unmanageable, so I was asked to administer pentothal. When 7 c.cm. of a 5 per cent. solution had been injected, respiration ceased and the patient became cyanosed. Without considering that this dose would be unlikely to cause respiratory paralysis, I attempted to inflate the lungs with oxygen. This was not successful, so a laryngoscope was passed and a small piece of food was seen in the pharynx. An intra-tracheal tube was passed, and suction removed more food, but the trachea still remained occluded. Tracheotomy was therefore performed, a tube was passed into the left bronchus, and a piece of onion was sucked out. The lungs were again inflated with oxygen and respiration was re-established. Auscultation shewed that the right bronchus was still blocked, and this in turn was sucked clear. The patient was evacuated next day and his subsequent history is not known.

**Case II** was similar to Case I except that when respiration ceased I was able to suck the food out of the trachea without performing tracheotomy.

**Case III.**—Four days after operation for appendicitis, a Dutch officer began to bleed briskly from the wound. He was quickly taken to the theatre ; the orderly who brought him stated that the patient had not had his supper. After only a few c.cm of pentothal solution had been injected the patient became cyanosed. A laryngoscope was passed, and soup was seen in the pharynx and also welling out of the trachea. It was sucked out, respiration was restored, and the bleeding point secured.

**Case IV.**—This was interesting as the patient had a compound fracture of the femur. He had some tea just after the accident: then travelled from Bourg Leopold in Belgium to Eindhoven in Holland and was brought on the operating table about three and a half hours after the accident. After a few c.cm. of pentothal had been injected the patient became cyanosed, and the tea was found in the trachea. After the operation the patient affirmed that he had had no tea on his arrival in hospital.

The points of importance in these cases are :—

1. There is no sign of vomiting. The food passes up the oesophagus and is then inhaled *quietly* into the trachea.

2. The absence of coughing.

3. As the patient becomes cyanosed quietly one is apt to assume that the cause is depression of the respiratory centre, and so to proceed to inflate the lungs without first establishing that the airway is clear.

4. It is essential to have a laryngoscope and suction apparatus at hand when using pentothal for an unprepared patient. The cause of these accidents was the slowness of the injection of the pentothal. All four of these cases occurred during the time the C.C.S. was receiving numbers of casualties, of which a high proportion were suffering from severe degrees of shock. As I was continually anaesthetizing shocked patients, I developed a habit of giving pentothal very slowly and carefully, as it is well known that the severely wounded man needs a very small dose to take him into the third stage. Now these four patients were not suffering from shock and the injection given slowly did not paralyse the vomiting centre.

After these accidents I never gave an unprepared patient who was not shocked less than 0.75 gm. in 5 per cent. solution, and I gave it quickly. Since then no further cases occurred. Before advising this method one must take great care to estimate the degree of shock. Professor Lundy, in his *Clinical Anaesthesia,* states that a full stomach is a definite contra-indication to the administration of an intravenous anaesthetic. While I agree with this abstention in civilian practice, it was not feasible in war surgery.

